# Development and internal validation of a diagnostic prediction model for life-threatening events in callers with shortness of breath: a cross-sectional study in out-of-hours primary care

**DOI:** 10.3399/BJGP.2024.0538

**Published:** 2025-05-07

**Authors:** Michelle Spek, Roderick P Venekamp, Anne AH de Hond, Esther de Groot, Geert-Jan Geersing, Anna SM Dobbe, Mathé Delissen, Frans H Rutten, Maarten van Smeden, Dorien L Zwart

**Affiliations:** Department of General Practice & Nursing Sciences, Julius Center for Health Sciences and Primary Care, University Medical Center Utrecht, Utrecht University, Utrecht; Department of General Practice & Nursing Sciences, Julius Center for Health Sciences and Primary Care, University Medical Center Utrecht, Utrecht University, Utrecht; Department of Epidemiology, Julius Center for Health Sciences and Primary Care, University Medical Center Utrecht, Utrecht University, Utrecht; Department of General Practice & Nursing Sciences, Julius Center for Health Sciences and Primary Care, University Medical Center Utrecht, Utrecht University, Utrecht; Department of General Practice & Nursing Sciences, Julius Center for Health Sciences and Primary Care, University Medical Center Utrecht, Utrecht University, Utrecht; Department of General Practice & Nursing Sciences, Julius Center for Health Sciences and Primary Care, University Medical Center Utrecht, Utrecht University, Utrecht; Department of General Practice & Nursing Sciences, Julius Center for Health Sciences and Primary Care, University Medical Center Utrecht, Utrecht University, Utrecht; Department of General Practice & Nursing Sciences, Julius Center for Health Sciences and Primary Care, University Medical Center Utrecht, Utrecht University, Utrecht; Department of Data Science & Biostatistics, Julius Center for Health Sciences and Primary Care, University Medical Center Utrecht, Utrecht University, Utrecht; Department of General Practice & Nursing Sciences, Julius Center for Health Sciences and Primary Care, University Medical Center Utrecht, Utrecht University, Utrecht

**Keywords:** dyspnoea, family medicine, general practice, out-of-hours primary care, prediction model, shortness of breath, telephone triage, triage

## Abstract

**Background:**

Shortness of breath often prompts calls to out-of-hours primary care and is the prime reason for home visits by GPs. However, a diagnostic prediction model in this setting is lacking.

**Aim:**

To develop and internally validate a model predicting life-threatening events for out-of-hours primary care callers with shortness of breath.

**Design and setting:**

This cross-sectional study includes data from 1952 patients with shortness of breath who called Dutch out-of-hours primary care between September 2020 and August 2021. The trial was registered with the Netherlands Trial Register, number: NL9682.

**Method:**

Four logistic regression models were developed with life-threatening events as the outcome. The first model included age and gender (model 1) and then, successively, call characteristics (calling at night and someone else calling on behalf of the patient; model 2), symptoms (cough, fever, inability to speak full sentences and wheezing; model 3), and medical history and medication use (cardiovascular and/or pulmonary; model 4) were added. The models were internally validated using optimism correction via bootstrap with 1000 repetitions. Performance measures of discrimination (c-statistic) and calibration (calibration intercept and slope) were determined.

**Results:**

Approximately 17% (329/1952) of callers with shortness of breath had a life-threatening event. Model 3 performed best. This model exhibited good discriminative ability (internal validation c-statistic of 0.76, 95% confidence interval = 0.74 to 0.79) and was well calibrated. All models had a high net benefit compared with using no model. Models 3 and 4 had a higher net benefit compared with models 1 and 2. As models 3 and 4 were similar in terms of net benefit, the model with fewer parameters (model 3) is preferred.

**Conclusion:**

A prediction model consisting of age, gender, call characteristics, and symptoms holds promise for improving telephone triage of callers to out-of-hours primary care with shortness of breath. However, as the study period coincided with the COVID-19 pandemic, temporal validation is necessary before widespread implementation.

## Introduction

Outside regular working hours, out-of-hours primary care provides urgent general practice care and ensures 24/7 medical access. In the Netherlands, similar to many other countries, out-of-hours primary care is organised in large-scale cooperatives.^1^ Under the supervision of a GP, triage nurses assess the urgency of a caller’s health problem by telephone, determining whether the patient requires attention from a GP or ambulance, the timeframe for this medical attention, and the type of contact needed (immediate ambulance, home visit, consultation with a GP, or telephone advice).^2^

In the Dutch healthcare system, GPs have a gatekeeping role, meaning that patients cannot directly access emergency departments without first consulting the out-of-hours primary care services. Patients need first to contact the out-of-hours primary care. However, lay people are allowed to contact the ambulance service directly on behalf of a patient when the situation is considered life-threatening. Then triage is performed by the ambulance personnel. Patients may be referred back to out-of-hours primary care if the ambulance personnel consider a direct ambulance dispatch unnecessary. On the other hand, the out-of-hours primary care service has the authority to dispatch an ambulance based on the telephone triage when a situation is considered potentially life-threatening. This structured approach helps to ensure that patients receive the appropriate level of care in a timely manner while also streamlining access to medical resources.

**How this fits in**

Shortness of breath (SOB) often prompts calls to out-of-hours primary care and is the prime reason for home visits by GPs. However, a diagnostic prediction model in this setting is lacking. A prediction model consisting of age, gender, calling at night, someone else calling on behalf of the patient, coughing, fever, inability to speak full sentences, and wheezing exhibited good discriminative ability for the outcome of life-threatening events and was well calibrated. After temporal validation of the current study’s prediction model, a decision scheme should be constructed to adapt telephone triage decision support systems to assign urgency based on the likelihood of a life-threatening event

Shortness of breath (SOB) often prompts calls to out-of-hours primary care and is the prime reason for home visits by GPs.^3^,^4^ SOB is the second most common reason for ambulance dispatches following calls to out-of-hours primary care, accounting for 8.1% of all ambulance dispatches in the Netherlands.^3^ A recent cross-sectional study conducted in Denmark further emphasises the significant workload associated with SOB in primary care, with 79.1% of patient contacts related to SOB necessitating a face-to-face consultation with a GP.^5^ Addressing this substantial workload necessitates effective triage, which is especially critical given the potential of underlying critical medical conditions in patients with SOB that can be easily missed in primary care, including acute coronary syndrome (ACS), acute heart failure, pulmonary embolism, and severe exacerbation of asthma or chronic obstructive pulmonary disease (COPD).^6^–^8^

Since 2011, the Netherlands Triage Standard (NTS) has been implemented in the Dutch out-of-hours primary care setting to assist triage nurses in the triage process.^1^,^9^ The NTS is a semi-automatic decision support tool comprising a hierarchically ordered computer algorithm. Triage nurses select one of 56 ‘entrance complaints’, including SOB, based on the suspected most severe symptom. SOB is such a severe symptom that it is always chosen if a patient reports it. Only chest discomfort has a higher severity level. After having chosen SOB as the entrance complaint the NTS-generated questions are used to get an impression of whether the patient:

1. coughs blood;
2. is drooling;
3. is seriously ill; and
4. has fever.

The NTS automatically generates an urgency level linked to a maximum response time. Urgency levels range from U1 (immediate deployment of ambulance) to U5 (telephone advice).^1^,^9^–^11^ The triage nurse and/or supervising GP may overrule the NTS-generated urgency level if they deem a different urgency level more appropriate.^10^

A recent study indicated suboptimal performance of telephone triage using the NTS. Under the assumption that a high-urgency allocation (U1/U2) is adequate for those who are found to have a life-threatening event and low urgency allocation (U3/U4/U5) for those who have a non-life-threatening event, this urgency allocation using the NTS exhibited a sensitivity of only 0.56 (95% confidence interval [CI] = 0.50 to 0.61) and specificity of 0.61 (95% CI = 0.58 to 0.63).^12^ Overruling the NTS’s urgency allocation by triage nurses and/or supervising GPs did not substantially positively affect the sensitivity (0.56 versus 0.54, *P* = 0.458) but slightly improved specificity (0.61 versus 0.65, *P*<0.001).

Unfortunately, prediction rules specific for callers with SOB in out-of-hours primary care or the primary care setting are lacking. Although several prediction rules exist for patients with acute SOB in the emergency department, these rules primarily rely on physical examination parameters and/or laboratory tests, rendering them unsuitable for telephone triage.^13^–^15^

The aim of this study was, therefore, to develop and internally validate a model for predicting life-threatening events in patients who call out-of-hours primary care for acute SOB. Ultimately, this prediction model will provide insight into variables that can be used in a triage tool for patients with SOB calling out-of-hours primary care, for example, resulting in the adaptation of the NTS in the Netherlands. In the longer term, this model can underpin triage tools with empirical evidence, potentially benefiting similar systems internationally.

## Method

### Study design

This study is part of the Opticall study, a multiple-methods study aimed at describing and improving the telephone triage of callers with SOB in Dutch out-of-hours primary care. The rationale and design of this study are published in a protocol paper.^16^ The objective of the current cross-sectional study was to develop and internally validate a prediction model for diagnosing life-threatening events in callers to out-of-hours primary care with acute SOB.

### Study population

In this cross-sectional study, data were included from adult patients who called two Dutch out-of-hours primary care centres with SOB between 1 September 2020 and 31 August 2021. Patients were included when the telephone conversation was for triage (for example, not a consultation with ambulance personnel) and when follow-up data about their final diagnosis could be retrieved from the patient’s own GP’s electronic health record (EHR).^17^,^18^ Patients whose triage conversation was either not traceable in the computer system or performed in a language other than Dutch or English were excluded.

### Data collection

Data were collected from both out-of-hours primary care and the patients’ own GPs. Patient and call characteristics, medical history, medication use, and symptoms were collected from call recordings and out-of-hours primary care EHRs. If a variable was not mentioned during the telephone triage conversation, it was labelled as missing. Data from call recordings were linked to follow-up data about final diagnosis and admission to hospital within 30 days of the contact with out-of-hours primary care from the patient’s own primary care EHR, which includes hospital specialist letters. The researchers who collected the data from out-of-hours primary care were blinded to the follow-up data from the patient’s own primary care EHR and vice versa.

### Outcome

The primary outcome was a final diagnosis of a life-threatening event (yes versus no) within 30 days of the contact with out-of-hours primary care, among patients with SOB.

Life-threatening events included the following diagnoses: pulmonary embolism, ACS, acute heart failure, transient ischaemic attack, stroke, sepsis, anaphylaxis, pneumothorax, subcutaneous emphysema, gastrointestinal bleeding, Takotsubo cardiomyopathy, perforated diverticulitis, respiratory insufficiency, and severe anaemia. Furthermore, a diagnosis of a pulmonary infection (COVID-19, pneumonia, and/or exacerbation of asthma/COPD) were classified as either mild to moderate, or severe, which in this latter case then also classified as a life-threatening event. Here, severe was defined as requiring hospital admission or supplemental oxygen administration at home within 24 hours of the out-of-hours primary care index contact.

The diagnosis of heart failure was classified as either chronic/stable or acute de novo/severe exacerbation of chronic heart failure (which in this latter case then also classified as a life-threatening event). Most of the life-threatening events were predefined. However, during the review of patient records, the authors encountered some diagnoses that were not initially anticipated, for example, gastrointestinal bleeding, intestinal ischaemia, and hypertensive crisis. Each patient’s case with such a diagnosis was discussed by an expert panel of experienced GPs (blinded to the urgency allocation) to determine if it should be classified as a life-threatening event or not.

### Sample size considerations

The study used the minimum sample size criteria for developing prediction models proposed by Riley *et al* and used the ‘pmsampsize’ package in R to calculate the sample size.^19^ Based on a life-threatening event prevalence of 16.9% (329/1952 in this study), a suspected c-statistic of 0.70, and a total number of 1934 participants, it was possible to evaluate 15 candidate predictors. This was sufficient to include the predictors of the pre-specified models, including age as a cubic spline function with four nodes, and an interaction term between age and gender.

### Candidate predictors

Candidate predictions were based on:

- age and gender;
- the current NTS triage criteria for the entrance complaint SOB;
- predictors used in prediction models in the out-of-hours primary care setting for other complaints associated with life-threatening events;
- prediction models for SOB in the hospital setting;
- clinical reasoning by experienced GPs; and
- data availability.^9^,^14^,^20^,^21^

### Missing data

Predictors with over 50% missing were excluded from consideration in the models (Supplementary Table S1). Variables were also excluded that were prevalent in <5% of the patients, which limited their added value in a prediction model; these were stridor and coughing blood.

The ‘missForest’ package in R was used to impute missing values in the remaining candidate predictors. This function employs a random forest trained on observed data to predict missing values.^22^ Recent studies show that it outperforms other frequently used imputation methods such as multiple imputation by chained equation.^23^ The ‘missForest’ function was executed with a maximum of 20 iterations and a forest consisting of 100 trees. Parallel processing was enabled.

### Data analysis

Baseline characteristics are described descriptively. For continuous variables means and corresponding SDs are shown. All categorical variables were dichotomised as women and men (gender) or as being present or absent (for example, having fever, yes or no).

Four logistic regression models were developed, with life-threatening events as the outcome. The first was a basic model with only patient characteristics (age and gender; model 1) and successively call characteristics (model 2), symptoms (model 3), and medical history and medication use (model 4) were added. The authors deliberately chose to add the variables in this order because patient characteristics and call characteristics will always be available, whereas symptoms, medical history, and medication use need to be asked for. Model 1 consists of gender, age as a cubic spline function with four knots on the 0.05, 0.35, 0.65, and 0.95 percentiles, as recommended by Harrell,^24^ and an interaction term between age and gender. Model 2 consists of model 1 plus two call characteristics: a) someone else calling on behalf of the patient and b) calling at night. Model 3 consists of model 2 plus four symptoms: a) fever, b) coughing, c) wheezing, and d) inability to speak full sentences because of SOB. Model 4 consists of model 3 plus a) cardiovascular disease and/or cardiovascular medication use and b) pulmonary disease and/or pulmonary medication use.

To correct for overfitting, all models were internally validated using optimism correction via bootstrap with 1000 repetitions. The performance of the internally validated models was evaluated in terms of the area under the receiver operating characteristic curve (c-statistic), calibration slope, and calibration intercept.^25^

The c-statistic is a measure of discrimination quantifying the distinction between low-and high-urgency individuals. The c-statistic ranges from 0.5 to 1, with higher values indicating superior discrimination. The calibration slope and intercept are measures of calibration that are considered good if the proportion of patients receiving a given risk score closely matches the actual risk score. For the calibration slope, 1 indicates perfect calibration and, for the calibration intercept, 0 indicates perfect calibration. The likelihood ratio test, with an alpha of 0.05, was used to determine which of these internally validated models performed best.

Decision curves were also constructed comparing the net benefit of models 1, 2, 3, and 4 on patient care within the clinical workflow. These decision curves plot the net benefit over a range of decision probability thresholds.^26^,^27^ The net benefit measures the number of true-positive classifications (patients with a life-threatening event receiving high urgency) penalised for false-positive classifications (patients without a life-threatening event receiving high urgency). A decision curve was also plotted for the current variables from NTS, excluding the variable ‘drooling’ because data were not available for this. It is known that in practice patients rarely experience drooling, and thus this variable contributes minimally to distinguishing patients with and without life-threatening conditions. Finally, a table was created showing the diagnostic accuracy (sensitivity, specificity, positive predictive value, and negative predictive value with corresponding 95% CIs) of the final model across various model-based risk thresholds.

All data analyses were performed with R version 4.3.2 (regression modelling strategies (‘rms’) package).^28^ The study is reported in accordance with the Transparent Reporting of a multivariable prediction model for Individual Prognosis or Diagnosis (TRIPOD) criteria.^29^

### Patient and public involvement

Patients were involved as advisors in this study.^30^ Advisory board meetings were organised for relevant stakeholders including patient representatives. During these meetings, the stakeholders were invited to provide input on the conduct of this study and the communication plans.

### Consent

A waiver was used for informed consent (this exception to informed consent has been described in the Declaration of Helsinki and is further specified in the Council for International Organizations of Medical Sciences guideline that contains a part about waiving informed consent).^31^,^32^ Personal data and research data were de-identified according to European General Data Protection Regulation.

## Results

In total, 1952 patients with SOB were included, with a mean age of 53.5 years (standard deviation 21.5) years, and 1078 (55.2%) were women (Table 1). Of the participants, 329/1952 callers (16.9%) had a life-threatening event, of which severe COVID-19 infection (116/1952, 5.9%), acute de novo/severe exacerbation of chronic heart failure (51/1952, 2.6%), severe COPD exacerbation (40/1952, 2.0%), and severe pneumonia (35/1952, 1.8%) were most common (Supplementary Table S2). Of patients with a life-threatening event, 54.1% (177/329) received a high-urgency level (U1 or U2). In 1842 patients (94.4%), the diagnosis of a life-threatening event was made within 3 days of contact with out-of-hours primary care and was clearly related to the out-of-hours primary care contact and not a new episode with new symptoms (data not shown).

**Table 1:** Baseline characteristics of 1952 callers who called out-of-hours primary care with SOB, stratified by life-threatening event (yes or no)

Characteristic	Total (*n* = 1952)	Life-threatening event (*n* = 329, 16.9%)	No life-threatening event (*n* = 1623, 83.1%)
**Patient characteristic**			
Age, years, mean (SD)	53.5 (21.5)	64.4 (18.4)	51.3 (21.4)
Men	874 (44.8)	167 (50.8)	707 (43.6)
Women	1078 (55.2)	162 (49.2)	916 (56.4)

**Call characteristics**			
Calling at night (12.00 am to 8.00 am)	365 (18.7)	73 (22.2)	292 (18.0)
Someone else called on behalf of patient (*n* = 1948)^a^	988 (50.7)	241 (73.5)	747 (46.1)

**Symptoms mentioned during the call**			
Coughing (*n* = 1379)^a^	937 (67.9)	175 (75.1)	762 (66.5)
Fever (*n* = 1335)^a^	371 (27.8)	82 (36.9)	289 (26.0)
Unable to speak full sentences (*n* = 1607)^a^	232 (14.4)	93 (37.1)	139 (10.3)
Wheezing (*n* = 1275)^a^	189 (14.8)	42 (20.2)	147 (13.8)

**Medical history and use of medication**			
Cardiovascular disease or medication (*n* = 1037)^a^	442 (42.6)	109 (63.4)	333 (38.5)
Respiratory disease or medication (*n* = 1213)^a^	722 (59.5)	130 (67.7)	592 (58.0)

**Urgency allocation (** ***n*** ** = 1935)** a			
High-urgency allocation (U1 or U2)	750 (38.8)	177 (54.1)	573 (35.6)
Low-urgency allocation (U3–U5)	1185 (61.2)	150 (45.9)	1035 (64.4)

Data are *n* (%) unless otherwise specified.

a For these variables there were missing data.

SD = standard deviation. SOB = shortness of breath.

**Figure 1:**
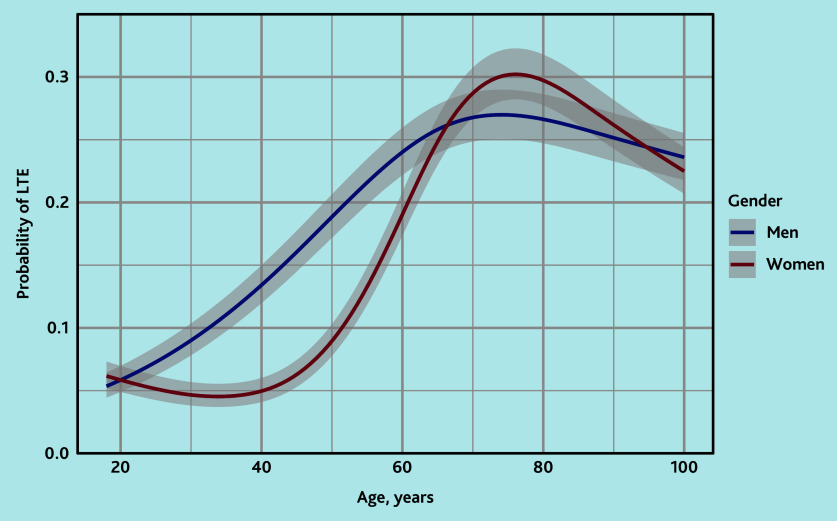
Logistic regression model with age and gender for predicting a diagnosis of a life-threatening event in patients calling out-of-hours primary care for shortness of breath. LTE = life-threatening event.

Patients with life-threatening events were on average older than those without life-threatening events. In men, the risk increased from 20 years onwards and stabilised around 75 years with a peak risk of 27% at the age of 74.3 years. In women, the risk increased from 40 years onwards and peaked at the age of 76 years with a 30% risk, after which the risk somewhat decreased again to a risk of around 27% (Figure 1).

The internal validation area under the curve of all four models is shown in Table 2. Model 3, consisting of age, gender, calling at night, someone else calling on behalf of the patient, coughing, fever, inability to speak full sentences, and wheezing, performed best according to the likelihood ratio test. The regression coefficients and odds ratios (ORs) of model 3 are shown in Table 3.

**Table 2:** Comparison of four prediction models for life-threatening events in patients calling out-of-hours primary care for SOB

Statistical measure	Model 1	Model 2	Model 3	Model 4
**AUC (95% CI)**	0.68 (0.65 to 0.71)	0.71 (0.68 to 0.74)	0.76 (0.74 to 0.79)	0.76 (0.74 to 0.79)
**Likelihood ratio test**	—	<0.01^a^	<0.01^b^	0.29^c^

a Likelihood ratio test comparing model 1 and model 2.

b Likelihood ratio test comparing model 2 and model 3.

c Likelihood ratio test comparing model 3 and model 4.

AUC = area under the curve. CI = confidence interval. SOB = shortness of breath.

**Table 3:** Final model (model 3) for predicting the diagnosis of a life-threatening event in patients callings out-of-hours primary care for SOB

Predictor	Regression coefficient (95% CI)	Odds ratio (95% CI)
Intercept	−4.52 (−6.39 to −2.65)	0.01 (0.00 to 0.07)
Age	0.04 (−0.02 to 0.10)	1.04 (0.98 to 1.10)
Age’	−0.02 (−0.18 to 0.15)	0.99 (0.84 to 1.16)
Age”	−0.03 (−0.44 to 0.39)	0.97 (0.64 to 1.48)
Female gender	1.91 (−0.65 to 4.47)	6.75 (0.52 to 87.48)
Interaction age and gender	−0.09 (−0.17 to −0.01)	0.92 (0.85 to 1.00)
Interaction age’ and gender	0.30 (0.06 to 0.54)	1.35 (1.06 to 1.71)
Interaction age” and gender	−0.74 (−1.34 to −0.13)	0.48 (0.26 to 0.88)
Calling at night (12.00 am to 8.00 am)	0.39 (0.07 to 0.71)	1.48 (1.08 to 2.03)
Someone else called on behalf of patient	0.50 (0.19 to 0.80)	1.65 (1.21 to 2.23)
Coughing	0.57 (0.26 to 0.88)	1.77 (1.30 to 2.40)
Fever	0.41 (0.11 to 0.71)	1.51 (1.12 to 2.03)
Unable to speak full sentences	1.19 (0.90 to 1.47)	3.27 (2.46 to 4.35)
Wheezing	0.28 (−0.09 to 0.65)	1.32 (0.92 to 1.91)

CI = confidence interval. SOB = shortness of breath.

The model performances of this model were: apparent c-statistic of 0.78 (95% CI = 0.75 to 0.80), internal validation c-statistic of 0.76 (95% CI = 0.74 to 0.79), internal validation intercept of −0.07 (95% CI = −0.24 to 0.16), and internal validation slope of 0.95 (95% CI = 0.84 to 1.08) (Table 4).

**Table 4:** Performance of internal validated final model (model 3) for predicting the diagnosis of life-threatening event in patients calling out-of-hours primary care for SOB

Model performance measure	Performance (95% CI)
**Apparent c-statistic**	0.78 (0.75 to 0.80)
**Internal validation c-statistic**	0.76 (0.74 to 0.79)
**Internal validation intercept**	−0.07 (−0.24 to 0.16)
**Internal validation slope**	0.95 (0.84 to 1.08)

CI = confidence interval. SOB = shortness of breath.

**Figure 2:**
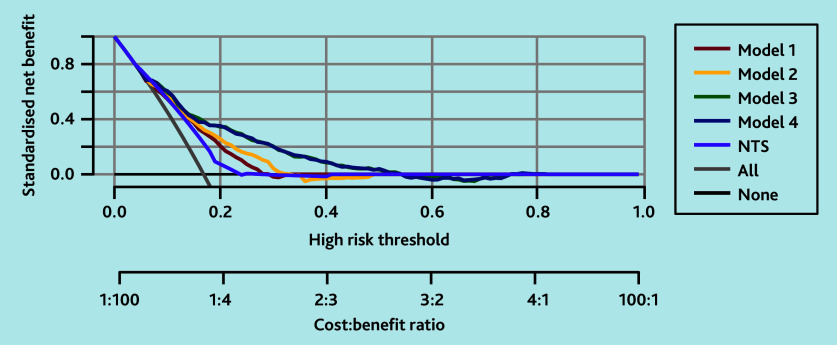
Logistic decision curve analyses plotting net benefit for models 1 to 4 for predicting a diagnosis of life-threatening event in patients calling out-of-hours primary care for shortness of breath. These decision curve analyses plot the net benefit for the four models compared with the current NTS criteria (except for drooling as data are missing for this variable) and compared with the baseline strategies of allocating high urgency to all (grey line) and allocating high urgency to none (black flat line). Models 3 and 4 demonstrated net benefit compared with NTS and the baselines across thresholds from 0.2, where each high-urgency allocation for a life-threatening event corresponds with four incorrectly high-urgency allocations for non-life-threatening events, to 0.4, where each two high-urgency allocations for life-threatening events correspond with three incorrectly high-urgency allocations for non-life-threatening events. NTS = Netherlands Triage Standard.

As shown by the decision curve analysis in Figure 2, all models had a high net benefit compared with using no model or the current NTS model for up to 0.4; at 0.4 each two high-urgency allocations for life-threatening events correspond with three high-urgency allocations for non-life-threatening events. Given the profound consequences of missing potentially life-threatening conditions, these lower thresholds are the most relevant for daily practice. So, the aim is to assign as much high urgency as possible to patients with a life-threatening event (safety), accepting that some patients without a life-threatening event will also be assigned high urgency ‘unnecessarily’ (inefficiency). Models 3 and 4 had a higher net benefit compared with models 1 and 2. As models 3 and 4 were similar in terms of net benefit, the model with fewer parameters (model 3) is preferred.

The diagnostic performance of the best prediction model (model 3) across risk thresholds for clinical application is presented in Table 5. When the threshold value is set at the prevalence rate of 17%, the sensitivity and specificity of the model are 0.69 (95% CI = 0.64 to 0.74) and 0.69 (95% CI = 0.67 to 0.72), respectively. At a lower threshold of 10%, the sensitivity increases (up to 0.89, 95% CI = 0.85 to 0.92) while the specificity decreases (0.48, 95% CI = 0.46 to 0.51).

**Table 5:** Diagnostic accuracy for a range of risk thresholds of the final model (model 3)

Risk threshold	Sensitivity (95% CI)	Specificity (95% CI)	PPV (95% CI)	NPV (95% CI)
**10%**	0.89 (0.85 to 0.92)	0.48 (0.46 to 0.51)	0.26 (0.23 to 0.28)	0.95 (0.94 to 0.97)
**17% (prevalence)** a	0.69 (0.64 to 0.74)	0.69 (0.67 to 0.72)	0.31 (0.28 to 0.35)	0.92 (0.90 to 0.93)
**25%**	0.54 (0.49 to 0.59)	0.82 (0.81 to 0.84)	0.38 (0.34 to 0.43)	0.90 (0.88 to 0.91)
**50%**	0.13 (0.09 to 0.16)	0.97 (0.97 to 0.98)	0.50 (0.39 to 0.61)	0.85 (0.83 to 0.86)

a The prevalence relates to the current study’s population.

CI = confidence interval. NPV = negative predictive value. PPV = positive predictive value.

## Discussion

### Summary

In this study a model for predicting life-threatening events in patients with acute SOB who called out-of-hours primary care, and in whom around 17% (329/1952) had a life-threatening event, was developed and internally validated. The best prediction model consists of age and gender, two call characteristics (calling at night and someone else calling on behalf of the patient) and four symptoms (coughing, fever, inability to speak full sentences, and wheezing). This model had a good discriminative ability and was also well calibrated.

All models had a high net benefit compared with using no model or the current NTS model for the lower thresholds. Models 3 and 4 had a higher net benefit compared with models 1 and 2. Models 3 and 4 outperformed the baselines for thresholds varying between 0.2, where each high-urgency allocation for a life-threatening event corresponds with four high-urgency allocations for non-life-threatening events, and 0.4, where each two high-urgency allocations for life-threatening events correspond with three high-urgency allocations for non-life-threatening events. In this range, it is particularly important for triage nurses to have a well-functioning model, as triage is especially challenging when some patients have the condition and others do not. If it is already clear that almost no one or almost everyone has the condition, a decision can be made without applying a model. As models 3 and 4 were similar in terms of net benefit, model 3 is preferred as it has fewer parameters. This ensures that the outcome maintains the same accuracy, while saving time by not requiring additional information on medical history and medication use. This efficiency allows for earlier involvement of the most appropriate help for the patient, which is especially important in patients with possible underlying life-threatening events.

The impact of this model on daily practice depends on the chosen threshold value. Given the profound consequences of missing potentially life-threatening conditions, the lower threshold values seem most relevant for daily practice. For the lower threshold values, the sensitivity of the model, as an indicator of safety, is substantially higher than that of the current NTS triage tool with only a limited impact on specificity, as an indicator of efficiency.

### Strengths and limitations

The current study, to the authors’ knowledge, is unique because the authors listened to the original triage conversations, which provided access to callers’ initial symptom presentation. These data were collected without knowledge about the final diagnosis, thus avoiding hindsight bias. Finally, the information from the back-up tapes was linked to follow-up data from the patient’s own GP, including specialist letters if the patient was referred, for a reliable determination of the final diagnosis up to 30 days after the contact with out-of-hours primary care.

Moreover, it was possible to include a large sample of patients without strict exclusion criteria, making the study population representative of the real-life situation. The approach used is therefore most likely generalisable to other countries with similar out-of-hours primary care services, including the UK, Germany, Scandinavian countries, and possibly others.^33^

It is distinctive that the study endpoint was a combination of diagnoses that can all be life-threatening. This is crucial for improving triage because, ideally, all life-threatening events should be assigned a high urgency. In the triage setting, it does not matter whether they are based on an ACS, pulmonary embolism, severe pneumonia, or another life-threatening event. Prediction models that focus on only one of these diagnoses would therefore be less suitable for application in telephone triage. Moreover, this model is especially useful for improving triage because it only used the variables available to the authors, namely patient characteristics, call characteristics, and symptoms mentioned by the caller. Despite this composite outcome, the model’s discrimination is good, and the CIs are relatively narrow, as clearly shown in Figure 1, indicating that prediction is nevertheless successful.

A limitation is the presence of missing values for several variables, particularly for symptoms not included in the current triage questions of the NTS entrance complaint SOB. The issue of missing data is common when using routine care data and is expected because of the nature of triage calls, as not all triage questions are asked of every patient. The current study had the benefit that the authors were able to re-listen to backed-up tapes. This made it possible to extract significantly more information and minimise missing values compared with those reported in the EHR of out-of-hours primary care. Importantly, the study employed appropriate imputation methods under the assumption that the data were missing at random.

The study period coincided with the COVID-19 pandemic, which potentially affected the results, as 5.9% (116/1952) of the study population had a severe COVID-19 infection and 21.1% (411/1952) had a mild or moderate COVID-19 infection. This should be considered when interpreting the findings, as it may render the study population less representative of the current patient population. Conversely, it is known that the clinical presentation of a severe COVID-19 infection is similar to that of community-acquired pneumonia with impending hypoxaemia.^34^ During the COVID-19 pandemic, other (severe) infections including severe pneumonia were displaced, suggesting that the current prevalence of life-threatening events is similar to that during the COVID-19 pandemic.^35^ Nevertheless, temporal validation of the prediction model, which is recommended in all developed prediction models, is therefore warranted.^36^

Another limitation is the absence of a universal list of conditions or severity of diseases that classifies for life-threatening. Consequently, the authors of the current study developed their own classification. As an example, the authors classified COVID-19 or pneumonia or exacerbation of heart failure only as a life-threatening event if the patient needed to be admitted to hospital within 24 hours. This classification was carried out by experienced GPs blinded to urgency allocation.

A definition of 30 days was used to ensure completeness and avoid missing cases; however, >94% (1842/1952) of patients were diagnosed within 3 days, indicating a clear presentation of acutely urgent medical conditions.

### Comparison with existing literature

It is difficult to compare the current findings with the literature because there are several prediction models for face-to-face triage in the emergency department but these items are not applicable for telephone triage and there is, to the authors’ knowledge, only one triage tool for SOB in primary care, the NTS, whose diagnostic accuracy has only very recently been studied and then found to be poor, while prediction models within this domain are lacking.^9^,^12^ This poor performance is problematic because it hinders safe and efficient telephone triage.^37^–^40^ Safe means that those who need urgent care receive it as soon as possible, in other words, to avoid ‘undertriage’ because this can lead to delay in diagnosis and treatment, and thus in some patients’ cases to irreversible harm or even death of the patient. Efficient means that those who do not need urgent care receive a less prompt medical evaluation, in other words, to avoid ‘overtriage’ and thus prevent disruption of the acute care chain. The latter is undesirable because it may cause delays for patients who truly need urgent care, and, in addition, it places an undesirable burden on healthcare resources, imposes unnecessary healthcare costs, may cause iatrogenic damage to the patient, and, finally, is associated with a potentially preventable climate impact of healthcare use.^10^,^11^

Comparison with prediction models from hospital settings is difficult because nearly all include physical examination parameters or laboratory tests that cannot be performed by telephone and sometimes even not at all in primary care settings.^13^–^15^ However, one of these studies has shown that a simple verbal numeric rating scale could be an effective method to assess the patient’s perceived degree of acute SOB in addition to laboratory tests.^14^,^41^ Although the authors of the current study did not have any information about the perceived degree of SOB, SOB severity in terms of the inability to speak full sentences was also found to be an important predictor of life-threatening events in this study (OR 3.27, 95% CI = 2.46 to 4.35).

Interestingly, prediction models for out-of-hours primary care are scarce in the literature. To the best of the authors’ knowledge, prediction models suitable for telephone triage in the out-of-hours primary care setting have been developed solely for the outcomes of ACS and sepsis.^20^,^21^ The prediction model for the outcome sepsis analysed which parameters from telephone triage were associated with sepsis-related mortality and intensive care unit admission.^21^ The sepsis model included age, as did the current model, but no other parameters that were applicable in the current situation of callers in which the entrance complaint SOB was chosen, such as the type of action or specific entrance complaints that were used in the sepsis model.

In terms of age, the model in the current study, along with the two other described models in the out-of-hours primary care setting, found not surprisingly that the likelihood of the outcome increased with advancing age.^20^,^21^ The study in which a prediction model for ACS was developed included callers to out-of-hours primary care for chest discomfort. In the ACS study the parameters age, gender, and calling at night were also included in their final model, similar to the current study.^20^ This shows that these predictors could potentially be relevant in multiple domains.

Regarding gender, the OR of 6.75 (95% CI = 0.52 to 87.48) for women is a bit misleading in the current study as men had a higher risk of life-threatening events, but this effect is covered in the interaction terms between age (as cubic spline function) and gender. The lower risk of life-threatening events in women is described in the literature as well; a study in daytime Belgian general practice care showed that men with SOB had a higher risk of immediate referral, hospital admission, and death than women.^42^ Although the study did not report on the final diagnosis, this suggests that men were more likely to have an urgent underlying medical condition.

Regarding calling at night, the higher likelihood of life-threatening conditions may be related to the circadian rhythm of cardiovascular diseases, with a notable peak in the early morning.^43^ Additionally, the relatively less frequent calls from patients with more benign conditions during the night, as they are less likely to be awakened or stressed-out by their symptoms, is likely also a contributing factor.

Regarding cardiovascular history, this seems to be a predictor for ACS with univariable analysis, but not multivariable, which is in line with the current study.^20^ A cardiovascular history is important to estimate the patient’s long-term risk, but it seems it is not so useful for diagnosing ACS in those with acute chest discomfort nor in patients with acute SOB who contact out-of-hours primary care.

Future research could also explore the role of self-triage and whether it specifically improves detection of life-threatening events in those with SOB if blended with the existing triage system in out-of-hours primary care.^44^

### Implications for research and practice

After temporal validation of the current prediction model, a decision scheme for the entrance complaint SOB should be constructed. This scheme could then be used to adapt telephone triage decision support systems, such as the Dutch NTS, to assign urgency based on the likelihood of a life-threatening event. The authors plan to develop this new decision scheme in collaboration with its users, triage nurses, supervising GPs, and patients/callers, through action research.

The next step will be to determine the applicability of this new triage rule for SOB by comparing its accuracy to the currently deployed triage rule. To this end, the modified decision scheme will first be piloted in a specific region and compared with the current telephone triage system. Ideally, then triage nurses would be asked to request and register both the symptoms of the current triage and those of the new decision model, to avoid the need for imputing missing values, as was necessary in the current study. A computer system in which all data can be easily registered is therefore essential for this process. If proven effective, this prediction model could be applied to other countries with similar out-of-hours primary care systems.^33^

A prediction model consisting of age, gender, calling at night, someone else calling on behalf of the patient, coughing, fever, inability to speak full sentences, and wheezing holds promise for improving telephone triage of callers to out-of-hours primary care with SOB. However, as the study period coincided with the COVID-19 pandemic, temporal validation followed by an impact study is necessary before considering widespread implementation.

## Supplementary Information



## Data Availability

The data can be made available for researchers whose proposed use of the data has been approved at request of the corresponding author, with a signed data-access agreement.
