# Supplementary material for: Development and internal validation of a diagnostic prediction model for life-threatening events in callers with shortness of breath: a cross-sectional study in out-of-hours primary care
Source: Br J Gen Pract. 2025 May 7;75(756):e500–8. doi: 10.3399/BJGP.2024.0538 (PMC12070295; doi:10.3399/BJGP.2024.0538)
Supplement: Supplementary file 1 [file BJGP.2024.0538_suppl.pdf]

Supplementary file

Table S1. Diagnoses of 1,952 patients calling out-of-hours primary care with shortness of breath

Life-threatening events	Number of patients	Percentage (%)
Cardiovascular disorders		
Acute coronary syndrome	14	0.7
Acute heart failure	51	2.6
Respiratory tract disorders		
Severe asthma exacerbation	13	0.7
Severe COPD exacerbation	40	2.0
Severe COVID-19 infection	116	5.9
Severe pneumonia	35	1.8
Other disorders		
Anaphylaxis	14	0.7
Pulmonary embolism	19	1.0
Sepsis	12	0.6
Other life-threatening events*	14	0.7
Non-urgent disorders		
Cardiovascular disorders		
Stable heart failure	46	2.4
Respiratory tract disorders		
Mild or moderate asthma exacerbation	123	6.3
Mild or moderate COPD exacerbation	100	5.1
Mild or moderate COVID-19 infection**	411	21.1
Mild or moderate pneumonia	87	4.5
Upper respiratory tract infection	113	5.8
Other disorders		
Hyperventilation/anxiety/stress	141	7.2
Shortness of breath due to (existing) cancer	36	1.8
Unspecified chest pain***	85	4.4
Unspecified shortness of breath****	216	11.1
Other non-urgent disorders*****	266	13.6

* Transient ischaemic attack, stroke, pneumothorax, subcutaneous emphysema, gastro-intestinal bleeding, Takotsubo cardiomyopathy, perforated diverticulitis, respiratory insufficiency due to reduced consciousness, severe anemia

** Proven (most cases) and suspected COVID-19 infections

*** Cardiac pathology unlikely after cardiologist's or GP's diagnostic work-up, including those with musculoskeletal chest pain

**** Cardiac or pulmonary pathology unlikely after cardiologist's, pulmonologists, or GP's diagnostic work-up

***** Amongst others: atrial fibrillation or atrial flutter, gastro-esophageal reflux, costal contusion/fracture, bronchitis or bronchial hyperreactivity, shortness of breath due to terminal phase, hay fever

Table S2. Variables excluded from data analysis due to high levels of missing data or low prevalence.

The reason for exclusion (missings or prevalence) is shown in bold

	Missings	Prevalence
Medical history and use of medication		
Smoking	**96.6%**	54.5%
Symptoms mentioned during the call		
Ankle oedema	**95.1%**	56.3%
Anosmia	**94.9%**	49.0%
Chest pain	**56.2%**	58.9%
Conjunctivitis	**96.8%**	44.4%
Coughing blood	44.8%	**4.5%**
Coughing sputum	**66.8%**	33.7%
Immobilisation	**95.6%**	68.2%
Malaise	**65.2%**	78.9%
Musculoskeletal pain	**89.2%**	80.%%
Palpitations	**92.0%**	63.1%
Rhinitis	**78.1%**	52.6%
Swollen calf	**97.9%**	12.2%
Throat complaints	**81.6%**	61.9%
Tingling sensations	**94.7%**	67.3%
Chest pain characteristics		
Chest pain when breathing	**91.3%**	80.5%
Pain onset <12 hours	**85.0%**	56.3%
Pain duration >15 minutes	**87.1%**	95.6%
Posture-specific chest pain	**98.2%**	80.0%
Radiation of pain	**88.7%**	58.2%
Severe pain (score >7 on VAS)	**94.9%**	27.0%
Shortness of breath characteristics		
SOB on exertion	**63.4%**	86.6%
Stridor	41.2%	**3.3%**
SOB: shortness of breath, VAS: Visual Analogue Scale		
